# A Case of Trichinellosis in a 14-Year-Old Male Child at Hawassa University Comprehensive Specialized Hospital, Hawassa, Sidama, Ethiopia

**DOI:** 10.1155/2021/3624687

**Published:** 2021-10-15

**Authors:** Abebe Melese, Musa Mohammed, Worku Ketema, Alemayehu Toma

**Affiliations:** ^1^School of Medicine, Hawassa University, Hawassa, Ethiopia; ^2^School of Medical Laboratory Sciences, Hawassa University, Hawassa, Ethiopia; ^3^School of Pharmacy, Hawassa University, Hawassa Referral Hospital, Hawassa, Ethiopia

## Abstract

**Background:**

Trichinellosis develops after ingestion of *Trichinella* cysts in pork meat. It is one of the most important parasitic human pathogens in the world. It is, however, underreported in part because none of the clinical manifestations are pathognomonic. The primary mode of transmission is ingestion of raw meat. Among the symptoms are muscle pain, swelling, and myopathy. High-grade fever and other systemic symptoms are not unusual. The hallmarks are ophthalmic and musculoskeletal manifestations, particularly conjunctival haemorrhage with periorbital edema and subungual splinter haemorrhage. Although the majority of infections are mild and asymptomatic, severe infections can result in enteritis, periorbital edema, and myositis. *Presentation of the Case*. A 14-year-old male patient from Oromia Region, Arsi Zone, West Arsi Zone, Bishan Guracha area, which is almost completely encircled by mountains, presented with a complaint of worsening easy fatigability and asymmetric right thigh enlargement lasting one month. The pertinent physical examinations on presentation were puffy face and eyes, and there was a 4 cm by 5 cm mass on the right lateral thigh with no overlying skin colour change, on-tender, and no discharge. Eosinophilia of 14% was noticed on the complete blood count. The definitive diagnosis of trichinellosis was made by muscle biopsy. He was then managed with albendazole and prednisolone and improved.

**Conclusion:**

Patients with periorbital edema, myositis, or eosinophilia should be evaluated for trichinellosis. Individuals who have these symptoms and a history of eating pork meat should be suspected of having trichinellosis. Before eating raw pork meat, it is recommended that it be cooked properly.

## 1. Introduction

Human trichinellosis, caused by *Trichinella spiralis,* is a common disease with a burden of about 10,000 people per annum [1, 2]. *T. spiralis* is transmitted to humans by the consumption of raw meat from wild or domestic animals, mainly pork [3, 4]. Cultural factors such as traditional food prepared from undercooked meat play an essential role in the distribution of trichinellosis in the community. Consumption of cooked meat and meat products can reduce the incidence of trichinellosis even though the life cycle of the causative agent is maintained among wild animals [5, 6].

In addition to *Trichinella spiralis* (which was formerly assumed to be the only species of this organism [7]), a new *Trichinella* taxonomy encompassing eight species has been created based on recent genetic diversity and zoogeographical and epidemiological results [8–15]. The presence or absence of an intramuscular collagen capsule divides all 12 identified taxa genetically and physiologically into two distinct clades [14, 15]. Today, they are genotyped using molecular techniques [12].

*Trichinella spiralis*, *Trichinella nativa*, *Trichinella britovi*, *Trichinella murrelli*, *Trichinella nelsoni*, *Trichinella genotype* T6 (T6), *Trichinella genotype* T8 (T8), *Trichinella* genotype T9 (T9), *Trichinella pseudospiralis*, *Trichinella papuae*, and *Trichinella zimbabwensis* are the current species list [10].

Following the ingestion of raw meat, infective microfilariae excyst and invade the intestine; after undergoing several molts, they become a mature adult worm and produce microfilaria. The adult worms live in the intestinal mucosa and persist for 4 to 6 weeks in humans [16]. Microfilariae invade the epithelial cells of the intestine and migrate to the skeletal muscle with the help of blood and the lymphatic system. The infected muscle cells develop into nurse cells that contain a collagen capsule. The microfilaria encapsulated in muscle fibers remains for months to years [3].

If the number of infective microfilariae is few, mostly infection is asymptomatic; however, if the number is large, it can cause gastroenteritis tract-associated signs and symptoms. Following acute infection, chronic *T. spiralis* infection may cause conjunctivitis, sweating, and decreased muscle strength that can last for several years [16, 17].

There are no reported disparities in the rates of trichinosis between males and females. Pregnant patients have milder trichinosis symptoms than nonpregnant patients, and stillbirths and abortions have been documented. Trichinosis manifestations are often worse in breastfeeding women than in nonlactating women [18, 19].

Males and females engage in sexual activity, and females begin to produce new generations 5 to 7 days after infection. Within a few weeks, an intestinally immune-mediated host response develops, and immune effector mechanisms impact the survival of female parasites, resulting in persistent adult worm evacuation [19].

Whilst clinical variations between people infected with different *Trichinella* species have been identified [17, 19], these differences could not be attributed to the pathogen species since the amount of infecting larvae ingested by each person was uncertain. *T. spiralis* infections, on the contrary, may be more severe than *T. britovi* infections, which could be owing to the fact that *T. britovi* females are less prolific [20]. *T. murrelli* appears to induce greater cutaneous responses and less facial edema than other species [21].

Nonencapsulated *T. pseudospiralis* appears to cause signs and symptoms that stay longer [19, 22, 23]. Nonetheless, because of the clear lack of knowledge about the infectious dose for each patient, these discrepancies should be viewed with caution [19].

According to a study conducted in rural areas of Vietnam, the majority of *Trichinella*-infected people were between the ages of 16 and 59 (80.4%), with females being two times more likely than males. According to other investigations, the infected individuals were adults between the ages of 41 and 50 (35.2%).

During the 2012 incident, only one 6-year-old child became infected. Males were infected at a higher rate (84.2%) than females (15.8%) [24]. This discrepancy could be attributable to the fact that this report was made available to patients during the outbreak, rather than a large-scale regional survey such as the study in Vietnam [25].

Thailand-based research patients as young as 10 years old and as old as 65 years old are relatively rare, although the majority of patients are between the ages of 35 and 44, and the disease struck more males than women between 1962 and 2003, with no significant sex difference between 2004 and 2006 [26]. Patients in the Lao People's Democratic Republic were mostly adults in their late twenties and thirties, with males and women afflicted nearly equally [27].

Following exposure to gastric acid and pepsin, the larvae are liberated from the cysts. They next infiltrate the mucosa of the small intestine, where they mature into adult worms (females 2.2 mm in length; males 1.2 mm). The small bowel has a four-week life span. The females release larvae after one week, which travel to striated muscles and encyst.

Both environmental and human behavior factors influence the dissemination of *Trichinella spiralis* and *Trichinella britovi* [28].

In both domestic and sylvatic contexts, *Trichinella* nematodes can still be found in a variety of hosts. *Trichinella* spp. transmission to humans has recently been attributed to wild animals rather than domestic animals in Europe. In addition, distinct prevalence ratios of *T. spiralis* and *T. britovi* were discovered in different geographical zones. *T. spiralis* was found to be more prevalent in the wild boar population in the western and central areas of Poland (70–85%) than in the eastern part (46–60%). *T. britovi* was common in the red fox population throughout the area, but it was most prevalent in the east [29].

Serology is used to confirm the diagnosis, which is usually based on clinical symptoms.

Enzyme-linked immunosorbent assays (ELISAs), indirect immunofluorescence, and latex agglutination are among the many serologic assays available. Serology is often reliable, and western blots can be used to confirm results [30, 31].

Antibody levels are not detectable until three weeks or more after infection, making them ineffective for early diagnosis. Furthermore, antibody levels have a little relationship to the severity of the clinical course. Antibody tests can remain positive for years after clinical symptoms have subsided. False-positive serologic test findings can be caused by various helminthic infections and autoimmune disorders.

Antigen assays have been developed, but their sensitivity is low: the assay was positive in 76 percent of patients with proven infection in one study [32].

Identifying larvae on muscle biopsy can help make a definitive diagnosis. This is not always necessary, but it might be helpful when there is a lot of doubt about the diagnosis [17]. Muscle biopsy yields the best results in symptomatic muscles and near tendon insertions. The muscle should be inspected following enzymatic digestion to liberate larvae, in addition to the usual histopathologic testing. A preparation of the unfixed muscle crushed between microscope slides can be used to view the specimen undigested.

Although morphology makes it difficult to distinguish *Trichinella* species, molecular genetic assays can help [15, 33].

A combination polymerase chain reaction (PCR) for the definitive differentiation of *Trichinella* species and genotypes has been developed, but it is not available on the market [15].

## 2. An Ethical Review

Written informed consent was obtained from the father of the patient for the publication of this case report after a letter of permission was gained from the Hawassa University Institutional Review Board (IRB).

### 2.1. Case Presentation

A 14-year-old male patient from Oromia Region, Arsi Zone, West Arsi Zone, Bishan Guracha area, which is almost completely encircled by mountains, presented with a complaint of worsening easy fatigability and asymmetric right thigh enlargement lasting one month. Prior to these issues, he had abdominal pain, diarrhoea, and high-grade fever that lasted a week. He then developed associated complaints of swelling around his face and eyes, a headache, and muscle pain, and upon further questioning, the patient's father revealed that the entire family had eaten raw pork meat 40 days prior during their holiday celebration, but none of them had developed these symptoms except this child.

The pertinent physical examinations on presentation were as follows.

General appearance: puffy face.

Head, eye, ear, neck, and throat (HEENT): pink conjunctiva and periorbital edema.

Musculoskeletal: there was a 4 cm by 5 cm mass on the right lateral thigh with no overlying skin colour change, on-tender, and no discharge.

Ophthalmic unit evaluation: puffy eyes with conjunctival haemorrhage.

The complete blood count (CBC) was performed, and it revealed a white blood cell (WBC) count of 12000∗10^3^ with a differential of neutrophils 53%, lymphocytes 33%, and eosinophilia 14%.

Urine analysis and stool examinations were inconclusive. The renal and liver functions were checked, and they were all within normal limits. However, the lactate dehydrogenase (LDH) level was three times higher than normal. The serum creatinine kinase level was also four times higher than normal. Thigh ultrasound revealed soft tissue swelling, and the managing team decided to perform a muscle biopsy, it was performed, and the results were read by two senior pathologists, and the report was as follows.

**Figure 1:**
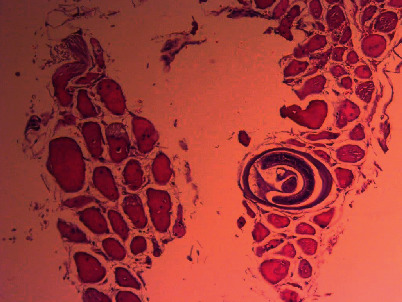
Cysts of *Trichinella spiralis* (low-power view).

**Figure 2:**
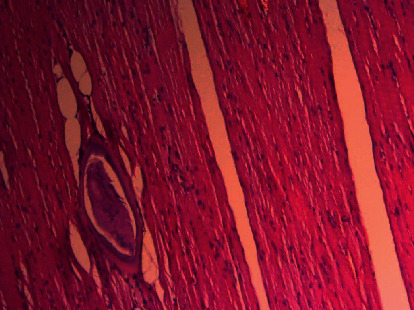
Encysted *Trichinella spiralis* (low-power view).

**Figure 3:**
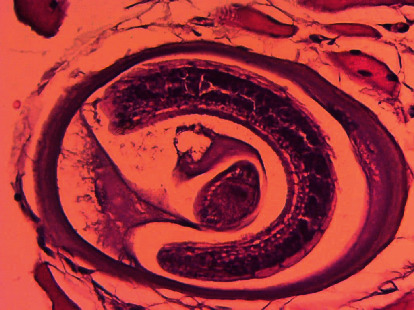
Cysts of *Trichinella spiralis* (high-power view).

It revealed skeletal muscle fragments composed of variable sized muscle fibers with intervening eosinophilic rich mononuclear cell infiltrates around calcified larvae with partial atrophy and destruction and well-encapsulated *Trichinella* parasites in muscle fibers (nurse cell), with the diagnosis of right lateral thigh parasitic myopathy (trichinellosis), as shown in Figures 1–3.

He was then treated with the recommended first-line medications, albendazole 400 mg bid for 14 days and prednisolone 30 mg po bid for 14 days.

After one month, he was reappointed and appeared to be healthy at the follow-up clinic, and the family was advised not to eat raw pork meat.

## 3. Discussion

Fever, eosinophilia, periorbital edema, and myalgia following a suspect meal can lead to a presumptive clinical diagnosis. The diagnosis is confirmed by an increase in the titer of the parasite-specific antibody, which usually occurs after the third week of infection.

Alternatively, a definitive diagnosis requires surgical biopsy of at least 1 g of the involved muscle; the yields are the highest near tendon insertions [34].

Clinical diagnosis of early and chronic trichinellosis is challenging as there are no pathognomonic signs and symptoms [21, 35]. Failing to diagnose the disease early may lead to the establishment of larvae in the muscle tissue and capsule formation which is difficult to treat [21, 35]. The diagnosis of trichinellosis can be based on clinical findings and nonspecific and specific laboratory findings such as eosinophilia, muscle enzymes, detection of the antibody, detection of larvae in a muscle biopsy, and epidemiological tracking of the source of infection [17]. The molecular technique can be used for the identification of the *Trichinella* species or genotype [35, 36].

To effectively treat and prevent long-term damage of trichinosis, provision of anthelmintics should be initiated at an early stage of the infection [17]. The disease can be prevented by controlling the disease among domestic and wild animals, reducing the contamination in the food industry, and avoiding eating raw meat [37]. In addition, significant efforts have been made to produce protective vaccines against *T. spiralis*; however, none of them are available currently for use. The difficulty in producing an effective vaccine is related to the complicated antigenic components of the parasite [6].

The recommended first-line medications, albendazole 400 mg bid for 14 days and prednisolone 30 mg po bid for 14 days, were given for our case [34]. Antiparasitic therapy is effective in treating larvae that burrow into the gastrointestinal tract [38]. However, the benefits of antiparasitic therapy in the case of blood-borne larval invasion or encysting of the muscle are unknown; treatment after muscle invasion may fail to eliminate infective larvae from the muscle.

In severe cases, prednisone at a dose of 30 to 60 mg/day for 10 to 15 days may be administered concurrently [36, 38, 39].

Eating pork meat is not common in most parts of Ethiopia. There are limited data on trichinellosis in Ethiopia. A case of trichinellosis was reported from Gojjam, Ethiopia, about 20 years ago. The case was linked to the eating of wild boars [40].

## 4. Conclusion

Trichinellosis should be considered in patients who have periorbital edema, myositis, or eosinophilia. Individuals with these symptoms and a history of consuming pork meat should be suspected of having trichinellosis. Before eating raw pork meat, it is recommended that it be cooked properly.

## Data Availability

The data used to support the findings of this study will be available from the corresponding author upon request.
